# Renoprotective Effect of Human Umbilical Cord–Derived Mesenchymal Stem Cells in Immunodeficient Mice Suffering from Acute Kidney Injury

**DOI:** 10.1371/journal.pone.0046504

**Published:** 2012-09-27

**Authors:** Te-Chao Fang, Cheng-Yoong Pang, Sheng-Chun Chiu, Dah-Ching Ding, Rong-Kung Tsai

**Affiliations:** 1 Institute of Medical Sciences, Tzu Chi University, Hualien, Taiwan; 2 Nephrology, Department of Internal Medicine, Buddhist Tzu Chi General Hospital, Hualien, Taiwan; 3 School of Medicine, Tzu Chi University, Hualien, Taiwan; 4 Department of Medical Research, Buddhist Tzu-Chi General Hospital, Hualien, Taiwan; 5 Department of Obstetrics and Gynecology, Buddhist Tzu Chi General Hospital, Hualien, Taiwan; 6 Department of Ophthalmology, Buddhist Tzu Chi General Hospital, Hualien, Taiwan; Children's Hospital Boston/Harvard Medical School, United States of America

## Abstract

It is unknown whether human umbilical cord-derived mesenchymal stem cells (hUC-MSCs) can improve the renal function of patients suffering from acute kidney injury. Moreover, before beginning clinical trials, it is necessary to investigate this renoprotective effect of hUC-MSCs in a xenogeneic model of acute kidney injury. However, no previous studies have examined the application of hUC-MSCs to immunodeficient mice suffering from acute kidney injury. The objectives of this study were to examine whether hUC-MSCs could improve renal function in nonobese diabetic-severe combined immune deficiency (NOD-SCID) mice suffering from acute kidney injury, and to investigate the mechanism(s) for hUC-MSCs to improve renal function in this xenogeneic model. Early (3 hr) and late (12 hr) administrations of hUC-MSCs (10^6^ cells) were performed via the external jugular vein into NOD-SCID mice suffering from either folic acid (FA) (250 mg/kg body weight) or vehicle. The results showed that early administration of hUC-MSCs improved the renal function of NOD-SCID mice suffering from FA-induced acute kidney injury, as evidenced by decreased serum urea nitrogen and serum creatinine levels, as well as a reduced tubular injury score. The beneficial effects of hUC-MSCs were through reducing apoptosis and promoting proliferation of renal tubular cells. These benefits were independent of inflammatory cytokine effects and transdifferentiation. Furthermore, this study is the first one to show that the reduced apoptosis of renal tubular cells by hUC-MSCs in this xenogeneic model is mediated through the mitochondrial pathway, and through the increase of Akt phosphorylation.

## Introduction

Acute renal failure (ARF) is a common disease that accounts for 2%–15% of hospitalized patients. The clinical symptoms and signs of ARF manifest as a rapid loss of the ability of the kidneys to excrete wastes, concentrate urine, and maintain fluid and electrolyte homeostasis [1]. In pathophysiology, ARF may result from prolonged renal hypoperfusion and renal ischemia or nephrotoxic substances, and is associated with tubular cell death and shedding of cells into the tubular lumen, resulting in tubular blockage and further decreasing glomerular filtration. The overall mortality rate of patients with ARF is still high (approximately 50%–80%) despite major advances in pharmacologic therapy, intensive care, and renal replacement therapy [1], [2]. Therefore, a more potent therapeutic intervention for ARF to reduce mortality is imperative.

Our previous study showed that endogenous bone marrow (BM) cells could contribute to the renal tubular epithelial cell population and regeneration of the renal tubular epithelium by DNA synthesis after folic acid-induced acute kidney injury (AKI), although most (90%) of the renal tubular regeneration came from indigenous cells [3]. These results have also been supported by another study [4]. Recently, a stem cell-based treatment strategy has started to become a realistic option to replace or rebuild damaged organs and tissues. Stem cell therapy has been successfully applied using a variety of cell types, including mesenchymal stem cells (MSCs), BM cells, and human umbilical cord blood cells, to rescue organ damage in animal and human studies [5]–[9].

On the basis of studies of allogeneic BM MSCs as a cell source for stem cell therapy for acute tubular necrosis, several studies have shown that sorted BM MSCs can rescue nonirradiated mice from acute renal tubular damage caused by toxins or ischemia [10]–[14]; however, it is still debatable whether the beneficial effects of MSCs are primarily mediated via their differentiation into target cells [10], [11], or by complex paracrine actions [12]–[14]. BM MSCs are obtained from human bone marrow; however, aspiration of BM is an invasive procedure, and the numbers and differentiation capabilities of BM MSCs decline significantly with age [15]. Fetal MSCs are derived from fetuses, a source associated with considerable ethical problems for human application, making these cells difficult to obtain. Compared with BM MSCs and fetal MSCs, human umbilical cord-derived mesenchymal stem cells (hUC-MSCs) can be separated from discarded umbilical cord, which causes no harm to the donor, and is not ethically problematic [16]. Therefore, hUC-MSCs are a safe and accessible source for large quantities of stem cells in comparison to fetal MSCs and BM MSCs. The viability of umbilical cord as a stem cell source is supported by the reports of several studies, which have demonstrated that there are abundant MSCs in human umbilical cords [17]–[20]. Our recent study showed that hUC-MSCs could enhance neuroplasticity in a mouse model of cerebral ischemia, using cells isolated from Wharton's Jelly of the umbilical cord. We were able to show that these hUC-MSCs have multilineage potential and that, under suitable culture conditions, they are able to transdifferentiate, *in vitro*, into adipogenic and osteogenic lineages, and neural cells [21]. It is still unknown whether the application of hUC-MSCs can improve the renal function of patients suffering from AKI. Therefore, before beginning clinical trials, it is necessary to investigate this renoprotective effect of hUC-MSCs in a xenogeneic model of AKI. Until now, no studies have examined the application of hUC-MSCs in immunodeficient mice suffering from AKI. One recent study showed that hUC-MSCs improved the renal function of immunocompetent rats suffering from bilateral renal ischemia-reperfusion injury (IRI). However, the mechanisms for the beneficial effects shown in this previous study have not yet been elucidated [22]. For example, that study did not investigate the caspase cascade in apoptosis. As we known, there are two major pathways of caspase cascade in apoptosis: the death-receptor pathway (caspase-8 dependent), which is mediated by activation of death receptors, and the mitochondrial pathway (caspase-9 dependent), which is mediated by noxious stimuli (DNA injury or oxygen radicals) that ultimately lead to mitochondrial injury [23]. The objectives of the present study were to examine the possible therapeutic potential of hUC-MSCs to rescue immunodeficient mice from AKI and to investigate the possible mechanism(s) by which hUC-MSCs may improve renal function in this xenogeneic model.

## Results

### Immunophenotyping of human umbilical cord; Immunophenotyping and *in vitro* differentiation of hUC-MSCs

The results of immunophenotyping of human umbilical cord, immunophenotyping and *in vitro* differentiation of hUC-MSCs were the same as our previous study [21]. In brief, the cells of Wharton's Jelly showed a fibroblastic morphology with a homogenous deposition of extracellular matrix and immunostaining showed strong positive signals against CD13, CD29, CD44 and CD90. However, cells were not immunostained by antibodies against CD34, CD45 and CD117, indicating that these matrix tissues were not of hematopoietic origin. Additionally, these rapidly dividing cells were extensively expanded, and subsequent flow cytometry revealed that the cells were positive for CD13, CD29, CD44, CD49b, CD73, CD90, CD105, CD166 and HLA-ABC but negative for CD1q, CD3, CD10, CD14, CD31, CD34, CD45, CD49d, CD56, CD117 and HLA-DA. These observations indicate that cells isolated from Wharton's Jelly of human umbilical cord have the same surface markers as those of MSCs, which were consistent with observations of bone marrow MSCs [24]. The fourth passage cells of hUC-MSCs were seeded at a density of 5×10^3^ cells/cm^2^ in culture medium and showed *in vitro* differentiation to form osteocytes, adipocytes, and neuroglial cells by immunohistochemistry staining as our previous study [21].

### Effects of early and late administrations of hUC-MSCs on renal function and renal histology changes of NOD-SCID mice suffering from folic acid (FA)-induced AKI

**Figure 1:**
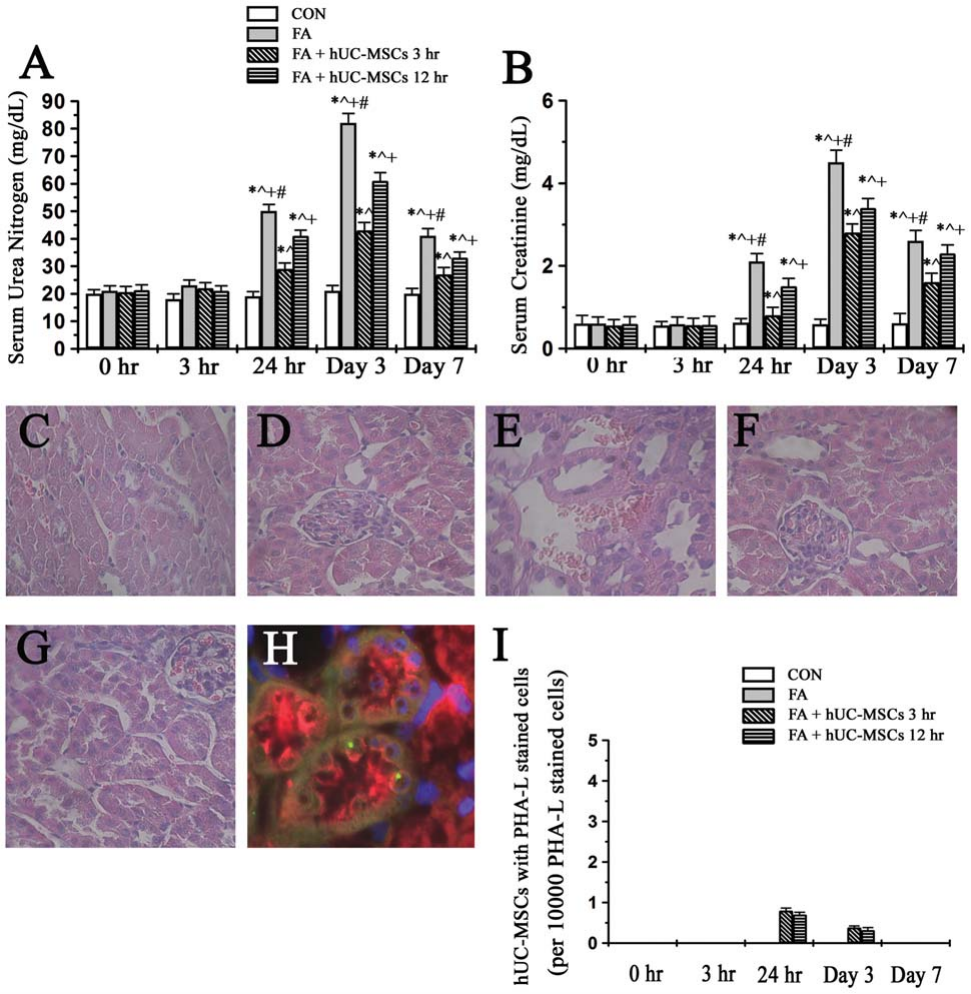
The effects of early and late administrations of hUC-MSCs on renal function (**Figure 1A and 1B**) and renal histology changes (**Figure 1C to F**igure 1I) of NOD-SCID mice suffering from folic acid-induced AKI. (A) The levels of serum urea nitrogen and (B) the levels of serum creatinine. NOD-SCID: nonobese diabetic-severe combined immunodeficiency disease; CON: control NOD-SCID mice; FA: NOD-SCID mice were treated with folic acid; FA+hUC-MSCs 3 hr: NOD-SCID mice suffering from folic acid and then given hUC-MSCs 3 hr later; FA+hUC-MSCs 12 hr: NOD-SCID mice suffering from folic acid and then given hUC-MSCs 12 hr later. Values are means ± SEM. *P<0.05 versus the same group at pre-folic acid period; ∧P<0.05 versus group CON at the corresponding time point; +P<0.05 versus group FA+hUC-MSCs 3 hr at the corresponding time point; #P<0.05 versus group FA+hUC-MSCs 12 hr at the corresponding time points. Additionally, (C) renal tubular cells were normal in the control period and (D) renal tubular cells were still unchanged at the time point of 3 hrs after folic acid administration. (E) Renal tubular cell damage appeared in folic acid-injured NOD-SCID mice at the time point of 24 hours after folic acid. (F) Early and (G) late administrations of hUC-MSCs to NOD-SCID mice suffering from folic acid could decrease renal tubular cell damage. Original magnifications ×400. (H) An example of engraftment hUC-MSCs (FITC-human chromosome X, green color) was observed in the renal tubular cells (*Phaseolus vulgaris* leucoagglutinin stained, red color) was seen. Original magnifications ×1000. (I) The engraftment of hUC-MSCs in the renal tubular cells was negligible at 24 hr and day 3 in folic acid-injured mice treated with early and late administrations of hUC-MSCs. Additionally, no engraftment of hUC-MSCs in the renal tubular cells could be detected at day 7.

Early administration (3 hr) of hUC-MSCs could have better renoprotection on mice suffering from FA-induced AKI than late administration (12 hr) of hUC-MSCs did (Figure 1A and 1B). Renal tubular cells were normal in control period (Figure 1C) and renal tubular cells were still normal at the time point of 3 hrs after folic acid administration (Figure 1D). Twenty-four hours after folic acid, renal tubular cell damage appeared in FA-injured NOD-SCID mice (Figure 1E). Additionally, early and late administrations of hUC-MSCs to NOD-SCID mice suffering from folic acid could decrease renal tubular cell damage (Figure 1F and 1G). An example of engraftment hUC-MSCs in the renal tubular was seen (Figure 1H). Although the engraftment of hUC-MSCs in renal tubules was seen at 24 hrs and day 3 in folic acid-injured mice treated with early and late administrations of hUC-MSCs (Figure 1I), the engraftment of hUC-MSCs was negligible (around 3∼8 cells in 10000 renal tubular cells). Additionally, no engraftment of hUC-MSCs in the renal tubular cells could be detected at day 7.

### hUC-MSCs improved renal function and renal histology changes of NOD-SCID mice suffering from FA-induced AKI

**Figure 2:**
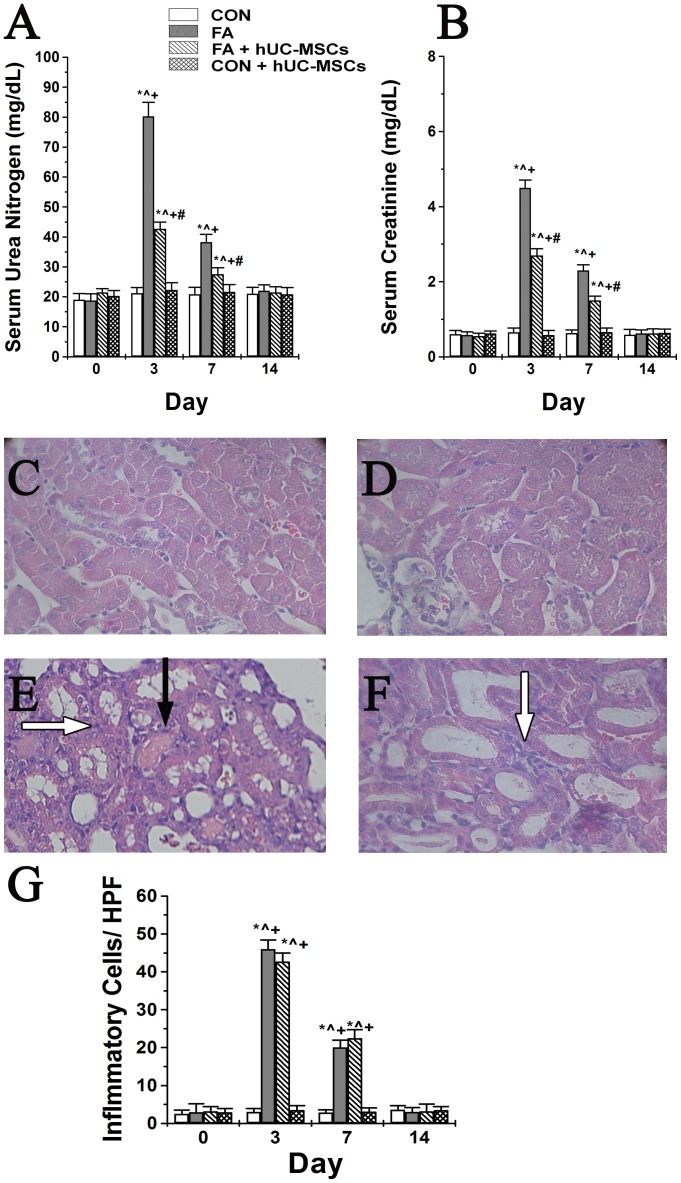
The effects of human umbilical cord-derived mesenchymal stem cells (hUC-MSCs) transplantation on renal function (A and B) and renal histology (C to G) of NOD-SCID mice treated with either folic acid or vehicle only. (A) The levels of serum urea nitrogen and (B) the levels of serum creatinine. NOD-SCID: nonobese diabetic-severe combined immunodeficiency disease; CON: control NOD-SCID mice; FA: NOD-SCID mice were treated with folic acid; FA+hUC-MSCs: NOD-SCID mice were treated with folic acid and then given hUC-MSCs 3 h later; CON+hUC-MSCs: NOD-SCID mice were treated with hUC-MSCs. Values are means ± SEM. *P<0.05 versus the same group at pre-folic acid period; ∧P<0.05 versus group CON at the corresponding time point; +P<0.05 versus group CON+hUC-MSCs at the corresponding time point; #P<0.05 versus group FA at the corresponding time points. Additionally, renal tubular cells were normal in the control period (C) and in control NOD-SCID mice with hUC-MSCs treatment throughout the experiment (D). Three days after folic acid, renal tubular cell damage was severe in folic acid-injured NOD-SCID mice (Figure 2E) and administration of hUC-MSCs to folic acid-injured NOD-SCID mice could decrease renal tubular cell damage (Figure 2F). The number of inflammatory cells in renal tubulointerstitial area per high power field was shown (Figure 2G). Black arrows indicate where some renal tubular epithelial cells were detached from the basement membrane (cell debris) into the tubular lumen, and white arrows indicate inflammatory cells in the renal tubulointerstitial area. Original magnifications ×100.

Figure 2 shows the effects of hUC-MSCs transplantation on the renal function and renal histology of NOD-SCID mice with and without FA administration. Serum urea nitrogen (SUN) and serum creatinine (SCr) levels showed a marked increase after FA administration, and both reached a peak at day 3, partially decreased by day 7, and returned to baseline levels by day 14 (Figure 2A and Figure 2B). hUC-MSCs treatment significantly reduced the levels of SUN and SCr in NOD-SCID mice treated with FA at day 3 and day 7 compared to controls. Renal tubular cells were normal in the control period (Figure 2C) and in the control NOD-SCID mice with hUC-MSCs treatment (Figure 2D) throughout the experiment. Three days after folic acid, renal tubular cell damage was severe in folic acid-injured NOD-SCID mice (Figure 2E) and administration of hUC-MSCs to folic acid-injured NOD-SCID mice could decrease renal tubular cell damage (Figure 2F). Additionally, folic acid treatment could induce inflammatory cell infiltration in the renal tubulointerstitial area when compared to control mice and control mice treated with hUC-MSCs (Figure 2G) at day 3 and day 7. However, there was no significant difference of inflammatory cell infiltration in the renal tubulointerstitial area between mice with folic acid injury and folic acid injured mice with hUC-MSCs treatment (Figure 2G) at day 3 and day 7.

Changes in tubular injury scores (TIS) are shown in Table 1. FA administration significantly increased TIS by day 3, which then decreased by day 7, and returned to baseline levels by day 14; this pattern concurred with that of changes in SUN and SCr. Additionally, hUC-MSCs treatment significantly reduced TIS after FA, and likewise SUN and SCr.

**Table 1:** Effects of administration of folic acid alone and in combination with human cord-derived mesenchymal stem cells on renal tubular injury scores in male NOD-SCID mice.

Tubular injury score		Pre-folic acid	Post-folic acid and hUC-MSCs		
	n		Day 3	Day 7	Day 14
Group CON	6	0.13±0.02	0.10±0.03	0.11±0.02	0.12±0.03
Group FA	6	0.12±0.02	2.78±0.06* ∧ +	1.52±0.04* ∧ +	0.11±0.03
Group FA+hUC-MSCs	6	0.11±0.03	1.32±0.04* ∧ + #	0.48±0.02* ∧ + #	0.13±0.02
Group CON+hUC-MSCs	6	0.12±0.03	0.10±0.02	0.11±0.03	0.12±0.02

CON: control; FA: folic acid; hUC-MSCs: human umbilical cord-derived mesenchymal stem cells. Values are the means ± SEM.

* P<0.05 versus the same group in the pre-folic acid period;

∧ P<0.05 versus group CON at the corresponding time point;

+ P<0.05 versus group CON+hUC-MSCs at the corresponding time point;

# P<0.05 versus group FA at the corresponding time point.

### Neither transdifferentiation nor fusion of hUC-MSCs was observed in renal tubular cells of NOD-SCID mice

**Figure 3:**
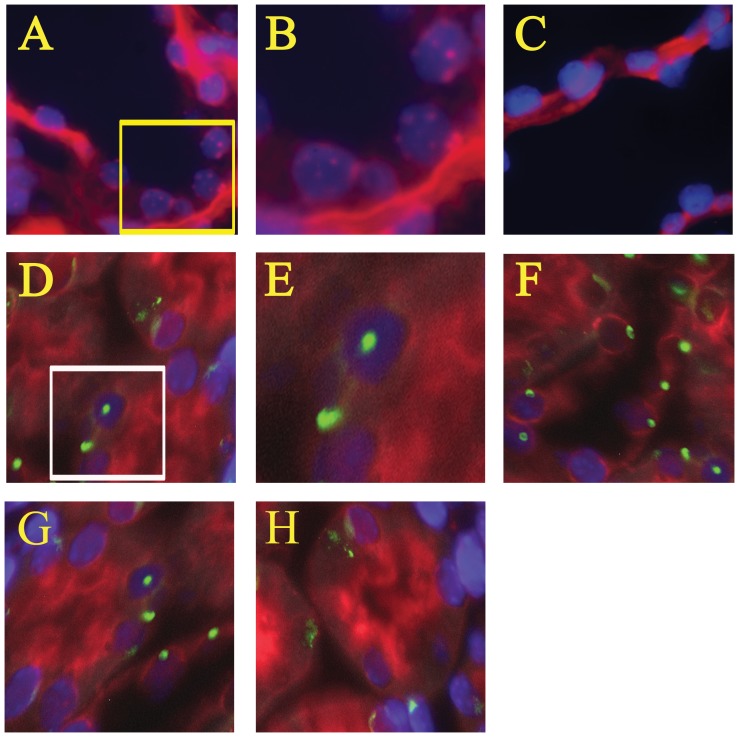
Examples of direct fluorescence *in situ* hybridization (FISH) examination to detect transdifferentiation or fusion of hUC-MSCs in PHA-L-stained renal tubular cells of folic acid (FA)-treated NOD-SCID mice with and without human cord-derived mesenchymal stem cells (hUC-MSCs). (A) human renal tissue (positive control for Cy3-labeled human pan-centromeric paint), (B) is a four-fold magnification of the yellow boxed area in A, (C) human renal tissue (negative control for Cy3-labeled human pan-centromeric paint), (D) normal NOD-SCID mice, (E) is a four-fold magnification of the white boxed area in D, (F) FA-injured NOD-SCID mice, (G) NOD-SCID mice were treated with folic acid and then given hUC-MSCs, (H) NOD-SCID mice were given hUC-MSCs. PHA-L stained renal tubular cells were stained as red color in the luminal side and cytoplasm. Direct FISH was performed using a Cy3-labeled human pan-centromeric paint (multiple red nuclear signals) and a FITC-labeled mouse Y-chromosome paint (a green nuclear signal) together in the same renal sections. DAPI identified nuclei and was seen as a blue signal. No signals of human pan-centromeric paint were observed in any PHA-L-stained cells among the four groups throughout the experiment. PHA-L: *Phaseolus vulgaris* leucoagglutinin; NOD-SCID: nonobese diabetic-severe combined immunodeficiency disease; DAPI: 4,6-diamidino-2-phenylindole. Original magnifications ×1000.

Direct fluorescence *in situ* hybridization (FISH) was performed to identify any transdifferentiation or fusion of hUC-MSCs with host murine renal cells using a human chromosome pan-centromeric paint and a mouse Y chromosome paint together in the same renal sections. However, rare signals of human chromosome pan-centromeric paint were observed in any *Phaseolus vulgaris* leucoagglutinin (PHA-L) stained tubular cells among the 4 groups throughout the experiment (Figure 3).

### hUC-MSCs did not modulate human inflammatory cytokine levels and mouse inflammatory cytokine levels in renal tissue of NOD-SCID mice suffering from FA-induced AKI

**Figure 4:**
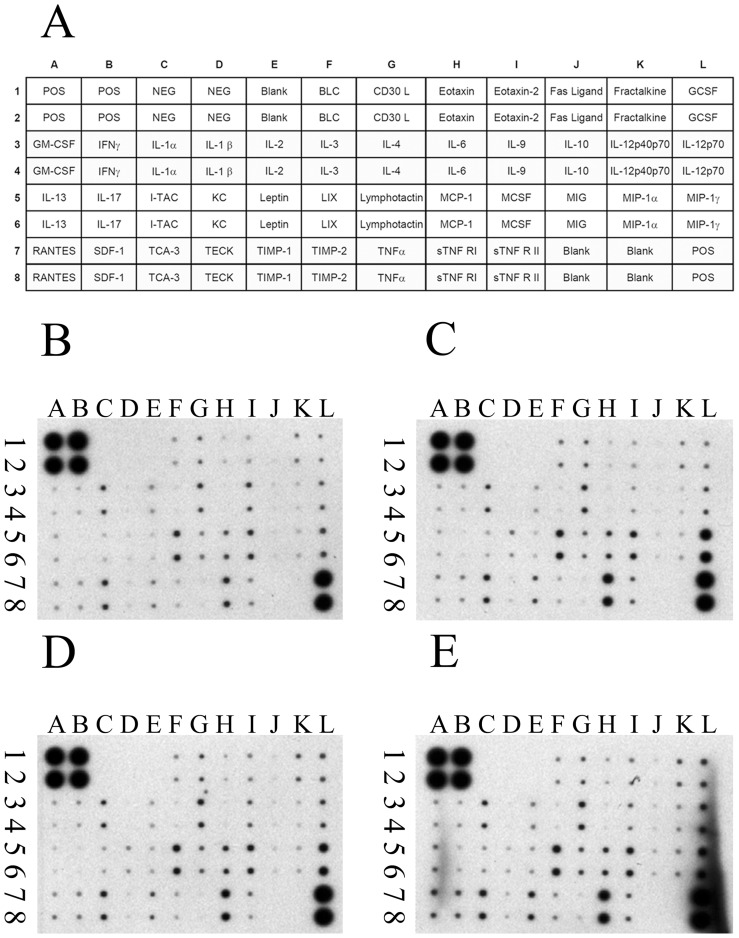
The effects of human umbilical cord-derived mesenchymal stem cells (hUC-MSCs) transplantation on cytokine expression, detected by a mouse cytokine array in the kidney tissue of NOD-SCID mice treated with either folic acid (FA) or vehicle only, at day 3 post-treatment. (A) Layout of the mouse cytokine array (RayBio mouse inflammation antibody array 1). Representative array readouts of mouse cytokine in the kidney tissue of (B) control NOD-SCID mice, (C) FA-injured NOD-SCID mice, (D) NOD-SCID mice were treated with folic acid and then given hUC-MSCs, and (E) NOD-SCID mice were given hUC-MSCs. The samples of renal tissue from each group were the pool of kidneys from six mice per group. The 40 mouse inflammation cytokines examined included B-lymphocyte chemoattractant (BLC), CD30 Ligand (CD 30 L), eotaxin, eotaxin-2, Fas Ligand, fractalkine, granulocyte colony-stimulating factor (G-CSF), granulocyte-macrophage colony-stimulating factor (GM-CSF), interferon (IFN)γ, interleukin (IL)-1α, IL-1β, IL-1ra, IL-2, IL-3, IL-4, IL-6, IL-9, IL-10, IL-12p40p70, IL-12p70, IL-13, IL-17, inducible T cell alpha chemoattractant (I-TAC), keratinocyte-derived cytokine (KC), leptin, lipopolysaccharide-induced CXC chemokine (LIX), lymphotactin, monocyte chemotactic protein (MCP-1), macrophage colony-stimulating factor (M-CSF), monokine induced by IFN-Gamma (MIG), macrophage inflammatory protein (MIP)-1α, MIP-1γ, RANTES (regulated on activation, normal T-cell expressed and secreted), stromal cell-derived factor-1 (SDF-1), T-cell activation protein 3 (TCA-3), thymus-expressed chemokine (TECK), tissue inhibitor of metalloproteinase (TIMP)-1, TIMP-2, tumor necrosis factor (TNF)α, soluble TNF Receptor I (sTNF RI), and soluble TNF Receptor II (sTNF RII). NOD-SCID: nonobese diabetic-severe combined immunodeficiency disease.

**Figure 5:**
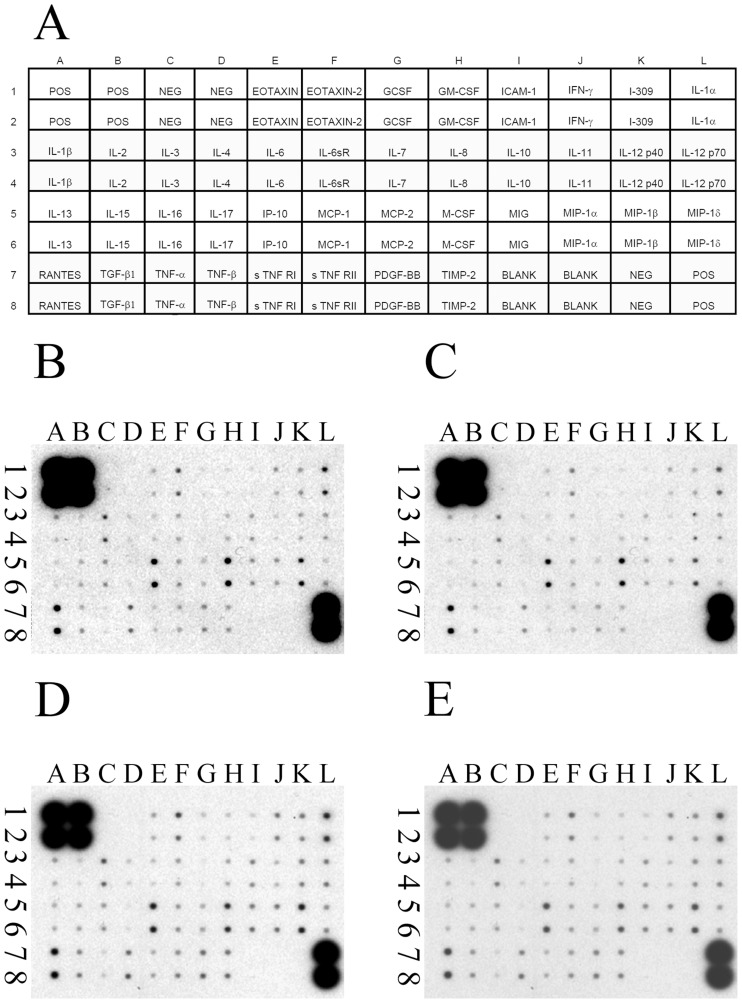
The effects of human umbilical cord-derived mesenchymal stem cells (hUC-MSCs) transplantation on cytokine expression, detected by a human cytokine array in the kidney tissue of NOD-SCID mice treated with either folic acid (FA) or vehicle only at day 3 post-treatment. (A) Layout of the human cytokine array (RayBio human inflammation antibody array 3). Representative array readouts of human cytokines in the kidney tissue of (B) control NOD-SCID mice, (C) FA-injured NOD-SCID mice, (D) NOD-SCID mice were treated with folic acid and then given hUC-MSCs, and (E) NOD-SCID mice were given hUC-MSCs. The samples of renal tissue from each group were the pool of kidneys from six mice per group. The 40 human inflammation cytokines examined included B-lymphocyte chemoattractant (BLC), eotaxin, eotaxin-2, granulocyte colony-stimulating factor (G-CSF), granulocyte-macrophage colony-stimulating factor (GM-CSF), I-309, inter-cellular adhesion molecule (ICAM)-1, interferon (IFN)γ, interleukin (IL)-1α, IL-1β, IL-1ra, IL-2, IL-4, IL-5, IL-6, IL-6 sR, IL-7, IL-8, IL-10, IL-11, IL-12p40, IL-12p70, IL-13, IL-15, IL-16, IL-17, monocyte chemotactic protein (MCP-1), macrophage colony-stimulating factor (M-CSF), monokine induced by IFN-Gamma (MIG), macrophage inflammatory protein (MIP)-1α, MIP-1β, MIP-1δ, platelet-derived growth factor B-chain homodimer (PDGF-BB), RANTES (regulated on activation, normal T-cell expressed and secreted), tissue inhibitor of metalloproteinase (TIMP)-1, TIMP-2, tumor necrosis factor (TNF)α, TNFβ, TNF soluble Receptor I (TNF sRI), and TNF soluble Receptor II (TNF sRII). NOD-SCID: nonobese diabetic-severe combined immunodeficiency disease.

To examine whether hUC-MSCs would affect the modulation of inflammation after FA treatment, human cytokine levels and mouse cytokine levels in renal tissues of experimental animals were analyzed via antibody array at day 3. The mouse proinflammatory cytokines (for example, IFN-γ, IL-1α, IL-1β, IL-6, and TNF-α) and mouse anti-inflammatory cytokines (for example, IL-4, -10, and -13) in mouse renal tissue were not significantly different among the 4 experimental groups (Figure 4). In addition, the human anti-inflammatory cytokines (for example, IL-4, -10, and -13) in mouse renal tissues were not significantly different between FA-injured NOD-SCID mice and FA-injured NOD-SCID mice treated with hUC-MSCs (Figure 5).

### hUC-MSCs enhanced tubular cell proliferation and reduced tubular cell apoptosis in NOD-SCID mice suffering from FA-induced AKI

**Figure 6:**
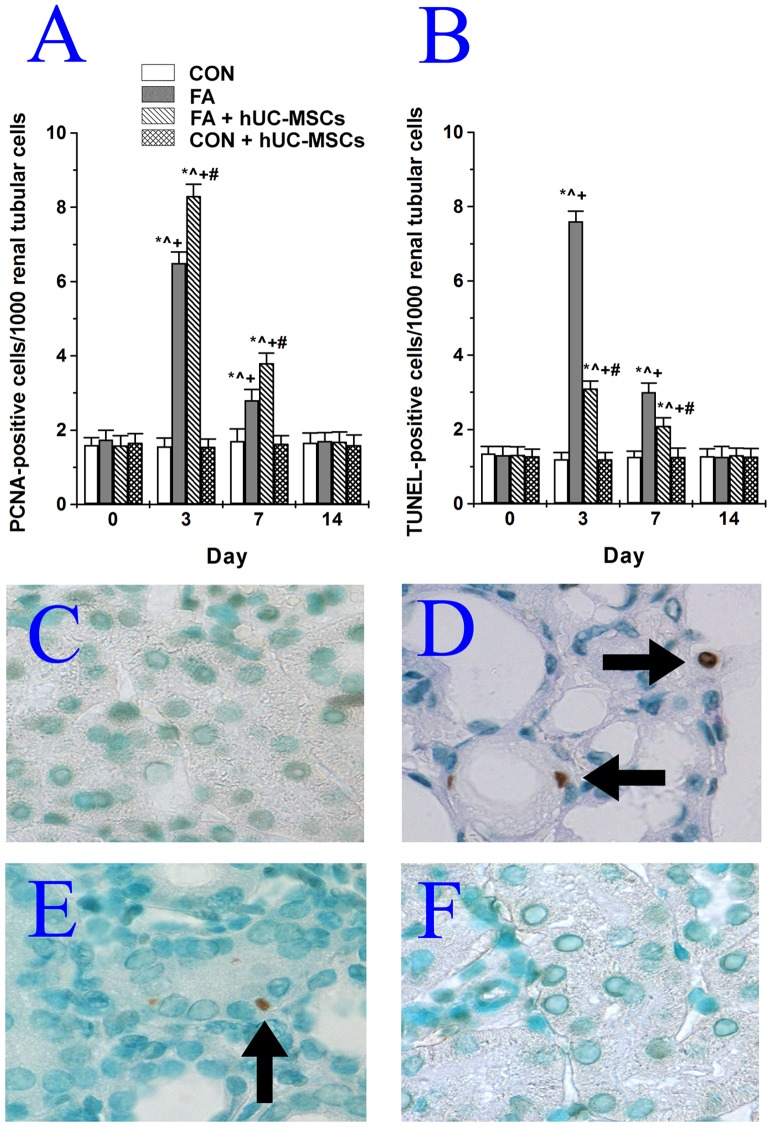
Effects of human umbilical cord-derived mesenchymal stem cells (hUC-MSCs) on tubular cell proliferation and apoptosis in NOD-SCID mice treated with folic acid. (A) Quantification of PCNA-positive tubular cells. (B) Quantification of TUNEL-positive tubular cells. NOD-SCID: nonobese diabetic-severe combined immunodeficiency disease; PCNA: proliferating cell nuclear antigen; CON: control NOD-SCID mice; FA: NOD-SCID mice were treated with folic acid; FA+hUC-MSCs: NOD-SCID mice were treated with folic acid and then given hUC-MSCs 3 h later; CON+hUC-MSCs: NOD-SCID mice were given hUC-MSCs. Values are means ± SEM. *P<0.05 versus the same group in the pre-folic acid period; ∧P<0.05 versus group CON at the corresponding time point; +P<0.05 versus group CON+hUC-MSCs at the corresponding time point; #P<0.05 versus group FA at the corresponding time point. Additionally, examples of stained TUNEL-positive cells (arrow) were seen in (C) control NOD-SCID mice, (D) NOD-SCID mice were treated with folic acid, (E) NOD-SCID mice were treated with folic acid and then given hUC-MSCs 3 h later, and (F) NOD-SCID mice were given hUC-MSCs.

The numbers of proliferating cell nuclear antigen (PCNA)-positive tubular cells and terminal transferase-mediated dUTP nick end labeling (TUNEL)-positive tubular cells significantly increased after FA administration, and both reached a peak at day 3, partially decreased by day 7, and returned to baseline levels by day 14 (Figures 6A and 6B). hUC-MSCs treatment significantly further increased PCNA-positive tubular cells from 6.5±0.3 to 8.3±0.3 (cells/1000 renal tubular cells) at day 3 and from 2.8±0.2 to 3.8±0.3 (cells/1000 renal tubular cells) at day 7 in NOD-SCID mice suffering from AKI by FA. However, hUC-MSCs treatment significantly decreased TUNEL-positive tubular cells from 7.6±0.3 to 3.1±0.2 (cells/1000 renal tubular cells) at day 3 and from 3.0±0.3 to 2.1±0.2 (cells/1000 renal tubular cells) at day 7 in NOD-SCID mice suffering from FA-induced AKI. Neither PCNA-positive tubular cells nor TUNEL-positive tubular cells were significantly different between control NOD-SCID mice and NOD-SCID mice treated with hUC-MSCs throughout the experimental period. Examples of TUNEL-positive tubular cells in NOD-SCID mice injured with FA and then treated with or without hUC-MSCs are shown in Figures 6C–6F.

### hUC-MSCs reduced tubular cell apoptosis in NOD-SCID mice suffering from FA-induced AKI through modulation of the mitochondrial pathway (caspase-9) but not the death receptor pathway (caspase-8) of apoptosis

**Figure 7:**
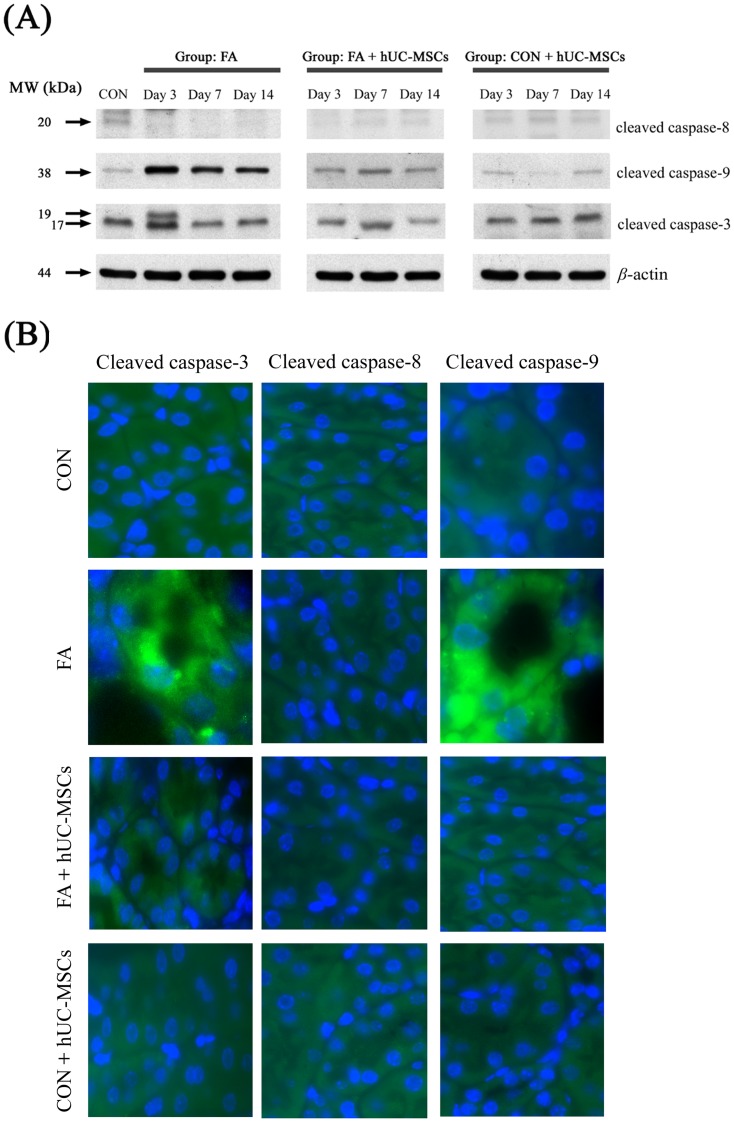
The effects of human umbilical cord-derived mesenchymal stem cells (hUC-MSCs) treatment on cleaved caspase-3, -8, and -9 expressions (assessed by western blots) in the kidney tissue of NOD-SCID mice treated with either folic acid or vehicle only (A) and examples of fluorescent immunohistochemistry staining for cleaved caspase-3, -8, and -9 in renal tubular cells of NOD-SCID mice treated with FA and subsequently either given or not given hUC-MSCs at day 3 (B). Folic acid injury increased the expression of the cleaved form of caspase-3 (19 KDa) and -9 (38 KDa), and subsequent treatment with hUC-MSCs decreased the expression of the cleaved forms of caspase-3 and -9. The sample size for each time frame per group was six mice and at least three independent experiments were performed. Abbreviations: NOD-SCID, nonobese diabetic-severe combined immunodeficiency disease; CON, control NOD-SCID mice; FA, NOD-SCID mice were treated with folic acid; FA+hUC-MSCs, NOD-SCID mice were treated with folic acid and then given hUC-MSCs 3 h later; CON+hUC-MSCs, NOD-SCID mice were given hUC-MSCs; MW, molecular weight; kDa, kilodalton.

To examine which pathway is involved in reducing tubular cell apoptosis following hUC-MSCs administration to NOD-SCID mice treated with FA, western blots were performed to measure active caspase-3 (common final pathway), active caspase-8 (death receptor pathway), and active caspase-9 (mitochondrial pathway) (Figure 7A). The expression of the cleaved form of caspase-9 (38 kDa) in the renal tissue of NOD-SCID mice treated with FA was increased compared to untreated controls, and the subsequent administration of hUC-MSCs decreased the expression of the cleaved form of caspase-9. The expression of a cleaved form of caspase-3 (19 kDa) in the renal tissue of NOD-SCID mice treated with FA was augmented at day 3, and subsequent administration of hUC-MSCs reduced the expression of this cleaved form of caspase-3. However, the expression of cleaved caspase-8 (20 kDa) was not significantly different among groups at any time period. Figure 7B shows an example of fluorescent immunohistochemistry staining for cleaved forms of caspase-3, -8, and-9 in renal tubular cells of NOD-SCID mice treated with FA and subsequently either given or not given hUC-MSCs at day 3.

### IGF1 secretion from hUC-MSCs and hUC-MSCs reduced tubular cell apoptosis in NOD-SCID mice suffering from FA-induced AKI through activation of the Akt phosphorylation

**Figure 8:**
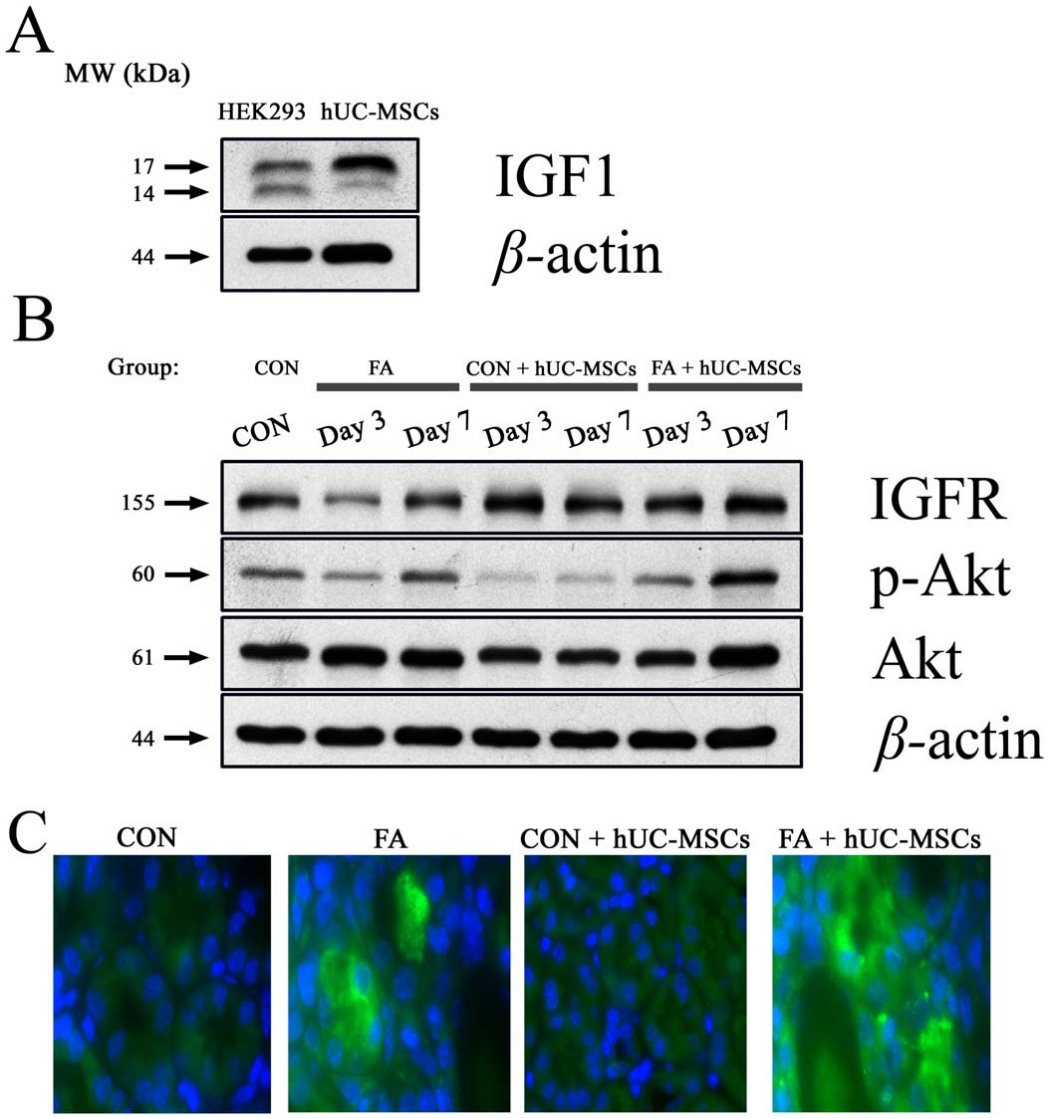
Western blot demonstrated the expression of IGF1 in the hUC-MSCs (A). HEK293, human embryonic kidney 293 cell line; hUC-MSCs, human umbilical cord-derived mesenchymal stem cells; IGF1, insulin-like growth factor 1. Additionally, IGF1 stimulated IGFR and then induced Akt phosphorylation in kidney cells of NOD-SCID mice which were suffering from folic acid and then given hUC-MSCs (B). At different time points, whole kidney extracts (made from the pool of kidneys from six animals per group) were used for Western blot analysis of IGFR, phsopho-Akt (Ser473-Akt), toal Akt, and β-actin (as internal control). The examples of phosphor-Akt expression in this kidney section were shown (C). IGFR, insulin-like growth factor 1 receptor; CON, control NOD-SCID mice; FA, NOD-SCID mice were treated with folic acid; FA+hUC-MSCs, NOD-SCID mice were treated with folic acid and then given hUC-MSCs 3 h later; CON+hUC-MSCs, NOD-SCID mice were given hUC-MSCs; MW, molecular weight; kDa, kilodalton.

The expression of IGF1, IGFR, p-Akt, and Akt were determined by Western blot analysis to examine whether the hUC-MSCs have an anti-apoptotic property through secretion of IGF1 and activation the IGFR, and then stimulation of the Akt phosphorylation. Figure 8A showed the expression of IGF1 by Western blot analyses of hUC-MSCs. Additionally, the expression of IGFR existed in the whole-kidney lysates of NOD-SCID mice with and without folic acid injury (Figure 8B). The ratio of phospho-Akt/Akt was increased after folic acid injury, and superimposed hUC-MSCs treatment on mice suffering from folic acid injury could further increase the ratio of phosphor-Akt/Akt (Figure 8B). We suggested that IGF1-derived from hUC-MSCs could stimulate IGFR and then induce Akt phosphorylation in the kidneys of NOD-SCID mice which were suffering from folic acid injury and then given hUC-MSCs. The examples of phosphor-Akt expression in renal sections were shown in Figure 8C.

## Discussion

The present study has demonstrated that (1) hUC-MSCs can improve the renal function of NOD-SCID mice suffering from FA-induced AKI; (2) administration of hUC-MSCs can reduce TIS and lower SUN and SCr in NOD-SCID mice suffering from AKI induced by FA compared to those of NOD-SCID mice treated with FA alone; and (3) the beneficial effects of hUC-MSCs are through increasing proliferation and decreasing apoptosis of renal tubular cells, and that these benefits are independent of modulation inflammatory cytokine effects and transdifferentiation. Moreover, this study is the first one to report that the reduced apoptosis of renal tubular cells following hUC-MSCs treatment in this xenogeneic model is mediated through the mitochondrial pathway, and through the increase of Akt phosphorylation.

### Cell transdifferentiation of hUC-MSCs

Although the engraftment of hUC-MSCs in renal tubules was seen at 24 hrs and day 3 in folic acid-injured mice treated with early and late administrations of hUC-MSCs, the engraftment of hUC-MSCs was negligible (around 3∼8 cells in 10000 renal tubular cells). At present, whether MSCs from humans can transdifferentiate into renal tubular cells in a xenogeneic model is still unclear. Our study showed that hUC-MSCs improved renal function of NOD-SCID mice suffering from FA-induced AKI, but these cells did not transdifferentiate into renal tubular cells, as rare signals of human pan-centromeres were detected in the kidney tissue of NOD-SCID mice. However, another study showed that administration of human BM MSCs improved renal function of immunocompetent rats suffering from glycerol-induced AKI, and that human BM MSCs transdifferentiated into renal tubular epithelial-like cells in this rat model [25]. The reason for the discrepant results between these two studies is unclear, and it may be due to the different origins (human BM and human umbilical cord matrix) of the MSCs, injury models (folic acid and glycerol-induced renal injury), or immunocompetency of the hosts. Similarly, whether MSCs from animals can transdifferentiate into renal tubular cells is still debatable [10], [12], [25]–[27].

### Effects of hUC-MSCs on modulation inflammatory cytokines

Previously, it was unclear whether hUC-MSCs could modulate inflammatory cytokines in renal tissue of NOD-SCID mice suffering from FA-induced AKI in a xenogeneic model. In this study, there were no significant changes in pro-inflammatory and anti-inflammatory cytokines (both human and mice) in renal tissues among groups at different time periods, although hUC-MSCs improved renal function in NOD-SCID mice suffering from FA-induced AKI. In contrast, several animal studies have demonstrated that allogeneic BM MSCs can facilitate renal function recovery in subjects (mice and rats) with AKI, and that the mechanism is cytokine dependent, but transdifferentiation-independent [14], [28], [29]. For example, an earlier study from Togel *et al.* showed that allogeneic BM MSCs could improve renal function in rats suffering from bilateral renal IRI. In this study, the expression of pro-inflammatory cytokines (IL-1beta, TNF-alpha, IFNgamma, and iNOS) was significantly reduced, and anti-inflammatory cytokines (IL-10, bFGF, TGF-alpha, and Bcl-2) were highly upregulated in the renal tissues of BM MSC-treated rats suffering from bilateral renal IRI. The beneficial effects of BM MSCs are primarily mediated via complex paracrine actions and not by their differentiation into renal tubular cells [14]. Similarly, Semedo *et al.* demonstrated that allogeneic BM MSCs improved renal function of rats suffering from bilateral renal IRI and the mechanism is dependent on a reduced expression of pro-inflammatory cytokines (IL-1beta, IL-6, and TNF-alpha) and an increased expression of anti-inflammatory cytokines (IL-4 and IL-10) [29]. The discrepant results of cytokine levels between this xenogeneic model and other allogeneic models may be due to the different immune statuses of the hosts (NOD-SCID mice and immunocompetent rats). It is well known that NOD-SCID mice manifest multiple functional defects of adaptive and innate immunity [30], including B and T cell deficiency, a functional deficit in NK cells, and impaired macrophage and complement functions [30], [31]. Therefore, there were no significant changes in pro-inflammatory cytokines and anti-inflammatory cytokines in the renal tissues of NOD-SCID mice suffering from FA-induced AKI. However, folic acid could induce the alternation of cell-death gene expression (Bcl2 and Bax) and the generation of oxidative stress, and the renal damage could have occurred at an earlier time point perhaps 6 hours after folic acid administration [32], [33]. Therefore, it is possible that the contribution of chemokines/cytokines at an earlier time point (before the peak of BUN) preceded the deterioration of renal function although we could not detect the significant differences at day 3. Because of this, we will investigate the chemokines/cytokines at earlier time points in the future.

### Effects of hUC-MSCs on promoting of proliferation and reducing of apoptosis

Our present study showed that injection of hUC-MSCs improved renal function in NOD-SCID mice suffering from FA-induced AKI, and promoted proliferation and reduced apoptosis of renal tubular cells. These effects are similar to those reported in other xenogeneic studies [22], [34]–[37]. For examples, human BM MSCs were found to attenuate AKI induced by cisplatin in an immunodeficient mouse model via decreased apoptosis and increased proliferation of renal tubular cells, although this study did not investigate transdifferentiation or cytokine effects [34]. Later, this study group demonstrated that human cord blood MSCs (hCB-MSCs) had a better survival rate than human BM MSCs in cisplatin-treated mice, and the mechanisms for hCB-MSCs to improve renal function of cisplatin-induced mice were by reducing apoptosis and by the rising in tubular cell proliferation [35]. Another study showed that hUC-MSCs improved renal function of immunocompetent rats suffering from bilateral renal IRI through increasing the percentage of PCNA-positive renal tubular cells, as well as by decreasing apoptosis (reducing caspase-3) of renal tubular cells, and promoting anti-inflammatory mechanisms (lowering the expression of interleukin-1beta) [37]. Taken together, MSCs derived from human umbilical cord blood [35] and human umbilical cord [22], [37] could improve renal function in animals suffering from AKI. However, the number of MSC in the human umbilical cord blood is low (around 0.00003%) [38], [39] and the human umbilical cord contained a very rich source of MSCs [40]. Therefore, obtaining MSCs after culture expansion from a piece of the human umbilical cord was easier. Nevertheless, the drawback of the culture expansion was the increased risk of contamination with any culture manipulation.

A recent study showed that hUC-MSCs improved renal function in immunocompetent rats suffering from bilateral renal IRI. In this previous study, hUC-MSCs treatment decreased caspase-3 and IL-1beta expression in kidney tissue compared with the control group, in addition to increasing the percentage of PCNA-positive cells [22]. No transdifferentiation was observed in this study [22]. However, this previous study did not explore which pathway of the caspase cascade in apoptosis might be influenced by hUC-MSCs. Our present study is the first one to demonstrate that injection of hUC-MSCs can improve renal function in NOD-SCID mice suffering from FA-induced AKI by modulating the mitochondrial and common pathways of apoptosis (reducing caspase-3 and -9, but not caspase-8), as well as promoting proliferation and decreasing apoptosis of renal tubular cells. It is reasonable to assume that there were no significant changes in serum tumor necrosis factor (TNF) cytokines in this immunodeficient model, and that it is impossible to active the death-receptor pathway because there are no members of the TNF superfamily to bind to cell surface “death receptors” (members of the TNF-receptor family) in this xenogeneic model. Furthermore, our study showed that IGF1-derived from hUC-MSCs could stimulate IGFR and then induce Akt phosphorylation in the kidneys of NOD-SCID mice which were suffering from folic acid injury and then given hUC-MSCs. A recent study also demonstrated that IGF1 could inhibit apoptosis through the activation of the Akt phosphorylation in pituitary cells [41].

In conclusion, we found that early administration of hUC-MSCs improved renal function in NOD-SCID mice suffering from FA-induced AKI, as evidence by a decrease in SUN and SCr, as well as a reduction in TIS. The beneficial effects of hUC-MSCs in this xenogeneic model were through reducing apoptosis and promoting proliferation of renal tubular cells, and these benefits were independent of inflammatory cytokine effects and transdifferentiation. Furthermore, this study is the first one to show that the reduced apoptosis of renal tubular cells following hUC-MSCs treatment in NOD-SCID mice suffering from AKI by FA is mediated through modulation of the mitochondrial pathway, and through the increase of Akt phosphorylation.

## Materials and Methods

### Experimental animals and protocols

The procedures for the animal experiments were carried out in adherence to the National Institutes Health Guide for the Care and Use of Laboratory Animals approved by the Institute of Animal Care and Use Committee of Tzu Chi Hospital. Nonobese diabetic-severe combined immune deficiency (NOD-SCID) (strain name: NOD.CB17-Prkdc^scid^/JTcu) mice were obtained from Tzu Chi University.

This study consisted of two experiments based on to determine whether the effects of early and late administrations of hUC-MSCs on renal function of NOD-SCID mice suffering from folic acid induced acute kidney injury and to examine the mechanisms of rescue for hUC-MSCs on this model. In the first study, six-week-old male NOD-SCID mice were randomly divided into 4 groups and treated as follows (*n* = 30 in each group): group CON, mice who received an intraperitoneal injection of vehicle (0.2 ml sterile 150 mM NaHCO_3_, Cat. No. S-6014, Sigma-Aldrich; St. Louis, MO, USA) only; group FA, mice received an intraperitoneal injection of folic acid (250 mg/kg body weight, Cat. No. F-7876, Sigma-Aldrich) in vehicle; group FA+hUC-MSCs 3 hr, mice were treated with folic acid (250 mg/kg body weight, intraperitoneal) in vehicle and given hUC-MSCs (10^6^ cells) by external jugular vein injection under IP ketamine (100 mg/kg) and xylazine (10 mg/kg) 3 hours after folic acid. Group FA+hUC-MSCs 12 hr, mice were treated as group FA and given hUC-MSCs (10^6^ cells) by external jugular vein injection under intraperitoneal ketamine (100 mg/kg) and xylazine (10 mg/kg) 12 hours after folic acid. The day of FA administration was designated as day 0. Mice were sacrificed before FA administration (0 hr), and on 3 hr, 24 hr, day 3, and 7 with 6 mice killed by overdose of pentobarbitone (Sagatal; Rhône Mérieux) at each time point. Terminal blood samples (0.7 ml) were obtained by cardiac puncture into an EDTA-tube. Bilateral kidneys were harvested and fixed in neutral buffered formalin before being embedded in paraffin wax for histological examination.

The preliminary results of first study showed early administration (3 hr) of hUC-MSCs could have better renoprotection on mice suffering from folic acid-induced AKI than late administration (12 hr) of hUC-MSCs did. Therefore, early administration of hUC-MSCs on the mice suffering from folic acid-induced AKI was performed. Six-week-old male NOD-SCID mice were randomly divided into 4 groups and treated as follows (*n* = 24 in each group): group CON, mice who received an intraperitoneal injection of vehicle (0.2 ml sterile 150 mM NaHCO_3_, Cat. No. S-6014, Sigma-Aldrich; St. Louis, MO, USA) only; group FA, mice received an intraperitoneal injection of folic acid (FA) (250 mg/kg body weight, Cat. No. F-7876, Sigma-Aldrich) in vehicle; group FA+hUC-MSCs, mice were treated with FA (250 mg/kg body weight, intraperitoneal) in vehicle and given hUC-MSCs (10^6^ cells) by external jugular vein injection under IP ketamine (100 mg/kg) and xylazine (10 mg/kg) 3 h after FA. Group CON+hUC-MSCs, mice were treated as group CON and given hUC-MSCs (10^6^ cells) by external jugular vein injection under intraperitoneal ketamine (100 mg/kg) and xylazine (10 mg/kg) 3 h after vehicle. The day of FA administration was designated as day 0. Mice were sacrificed before FA administration (day 0), and on day 3, 7, and 14, with 6 mice killed by overdose of pentobarbitone (Sagatal; Rhône Mérieux) at each time point. Terminal blood samples (0.7 ml) were obtained by cardiac puncture into an EDTA-tube. Left kidneys were harvested and fixed in neutral buffered formalin before being embedded in paraffin wax for later histological examination. Right kidneys were removed, decapsulated, minced, sonicated, and stored at −80°C within 1 h after collection for further analyses for inflammatory cytokines and western blotting (for caspase-3, -8, and -9).

### Preparation and immunophenotyping of hUC-MSCs via flow cytometry and immunohistochemistry

Protocols for sampling human umbilical cord were approved by the Research Ethics Committee of Buddhist Tzu-Chi General Hospital. Written informed consent was obtained from all mothers before labor and delivery of infants. Fresh human umbilical cord samples (20 cm in length, 20 g in weight) were obtained after delivery and collected in sterile boxes containing Hanks' balanced salt solution (Gibco/BRL 14185-052, USA) and separation of Wharton's Jelly from the vessels and amniotic membrane was performed within 24 h. The generation of therapeutically active hUC-MSCs had been successfully established by our previous study [21]. In brief, after the removal of blood vessels, the Wharton's Jelly was chopped into small pieces using scissors, then treated with collagenase type 1 (Sigma-Aldrich) and incubated for 14–18 h at 37°C in a 95% air/5% CO_2_ humidified atmosphere. Finally, the cells were washed and cultured in DMEM supplemented with 10% fetal bovine serum and antibiotics at 37°C in a 95% air/5% CO_2_ humidified atmosphere. The cultures were left undisturbed for 5–7 days to allow for migration of the cells from the explants. The cellular morphology became homogenously spindle shaped in cultures after 4–8 passages, and the specific surface molecules of these cells were characterized by flow cytometric analysis.

To demonstrate specific markers of MSCs in hUC-MSCs via flow cytometry, these cells were harvested using 0.5 mM EDTA solution. Cells were washed 3 times in PBS and adjusted to a concentration of 5×10^5^/200 µl. Cells were incubated with 1∶200 dilutions of primary antibodies including CD1q, CD3, CD10, CD13, CD14, CD31, CD34, CD45, CD90, CD73, CD56, HLA-ABC, HLA-DR, CD49b, CD49d, Cd29, CD44, CD105, CD117, and CD166 (BD Pharmingen) at 6–12°C for 30 min followed by 3 PBS washes. Cells were then stained by goat anti-mouse FITC secondary antibody at 6–12°C for 30 min followed by 3 PBS washes, adjusted to 1×10^6^ cells/ml, and analyzed using a flow cytometer (Becton Dickinson, San Jose, CA).

To demonstrate specific MSC antigens in hUC-MSCs via immunocytochemistry, hUC-MSCs were seeded overnight in 24-well culture plates (1×10^4^/well) and fixed with 10% formaldehyde for 20 min at 4°C. Nonspecific binding was blocked with normal serum from the species in which the secondary antibodies were raised (10% normal serum in PBS containing 0.25% Triton X-100). The fixed stem cells were incubated with the primary antibodies diluted in PBS containing 0.25% Triton X-100 for 2 hours at 25°C. The primary antibodies used in the study were as follows: anti-CD13, anti-CD29, anti-CD34, anti-CD44, anti-CD45, anti-CD90, anti-CD105, and anti-CD117 (dilution 1∶200; BD Pharmingen). After rinsing in PBS, the cells were incubated in the dark with FITC- or rhodamine-conjugated secondary antibodies for 1 h at room temperature and observed using an Axiovert 200M (Zeiss) inverted fluorescent microscope. Images were taken and processed with Metamorph (Universal Imaging Co, Ver 6.0 rev 5) equipped with a CoolSnap HQ CCD camera. In the controls, all procedures were processed in the same manner, except that the primary antibodies were omitted.

A clonal hUC-MSCs population was chosen for these experiments and was used at passage 4. Before injection into mice, cells were assessed to confirm their capability for trilineage differentiation *in vitro* as we previously reported [21], and their mesenchymal phenotype was also assessed by fluorescence-activated cell sorting (FACS) [21].

### SUN and SCr measurements

Blood samples were centrifuged at 5000× *g* for 10 min at 4°C and supernatants were kept at −70°C for later determination of SUN and SCr using a COBAS Integra 800 analyzer (Roche Diagnostics, Basel, Switerland). The levels of SUN and SCr were expressed in milligrams per 100 ml.

### Histology and semiquantitative morphometric analysis

For histological examination of renal tissue, 4-µm-thick sections were stained with periodic acid-Schiff. Tubular injury was assessed at ×400 total magnification using 20 consecutive and non-overlapping fields of view (adjacent to the corticomedullary junction) of periodic acid-Schiff stained specimens. Tubular damage, defined as tubular epithelial swelling, loss of brush border, vacuolar degeneration, necrotic tubules, and desquamation, was scored semiquantitatively. Tubular injury was categorized into 1 of 5 scores using the following criteria: 0, normal; 1, 2, 3, and 4 based upon the report by Nangaku *et al.* [42], [43]. The mean TIS for each mouse represented the average score of the 20 fields of view immediately adjacent to the corticomedullary junction.

Additionally, renal sections were statined with hematoxyline and inflammatory cell infiltration in the renal tubulointerstitial area was count in ten high power fields (HPF) per mouse, and the results were expressed as the inflammatory cells per HPF.

### Combination staining for renal tubular marker (PHA-L Lectin) and marker of hUC-MSCs (human pan-centromeric paint)

To examine if hUC-MSCs exhibited transdifferentiation or fusion in renal sections of NOD-SCID mice suffering from FA-induced AKI, renal sections were stained for PHA-L (by histochemical methods) and by FISH with human pancentromeric paint and mouse Y chromosome paint.

#### Treatment of sections

Sections cut at 4-µm thickness were dewaxed and incubated with 30% hydrogen peroxide in methanol to block endogenous peroxidase, then gradually rehydrated through a descending series of ethanol concentrations in water (100%, 95%, and 70%). Sections were stained for lectin (PHA-L) and were incubated in bovine trypsin (390414M; BDH Laboratory Supplies) (100 mg bovine trypsin, 100 mg CaCl_2_, 100 ml distilled water, pH 7.8) at 37°C for 15 min, then washed in PBS. All sections were incubated for 5 min in 20% acetic acid in methanol to block endogenous alkaline phosphatase activity.

#### Histochemical method for detection of proximal tubular epithelial marker

For the detection of lectin binding sites in kidney tissues, slides were incubated with the biotinylated PHA-L (B-1115; Vector Laboratories) 1∶100 in PBS for 45 min at room temperature. Sections were then washed in PBS and subsequently incubated with streptavidin-alkaline phosphatase (D0396; DAKO) for 30 min at room temperature. Following washes in PBS, Vector Red Substrate (SK 5100; Vector Laboratories) was applied for 8 min at room temperature and sections were again washed in PBS prior to the *in situ* hybridization protocol.

#### FISH for detection of hUC-MSCs in mouse kidney tissue

To determine whether hUC-MSCs could transdifferentiate into mouse kidney tissue, direct FISH using human pan-centromeric paint and mouse Y chromosome paint was performed.

Sections were incubated in 1 M sodium thiocyanate (S7757; Sigma-Aldrich) for 10 min at 80°C to improve access of the probes to DNA, washed in PBS, and then digested in 0.4% w/v pepsin (P6887; Sigma-Aldrich) in 0.1 M HCl for 10 min at 37°C to improve access of detection antibodies to the probes. The protease was quenched in 0.2% glycine (G4392; Sigma-Aldrich) in 2× PBS and sections were then rinsed in PBS, post-fixed in 4% paraformaldehyde (P6148; Sigma-Aldrich) in PBS, dehydrated through graded ethanols, and air dried. The probe mixture of Cy3-labeled human chromosome pan-centromeric paint (1695-CY3-01; Cambio) plus mouse FITC-labeled Y chromosome paint (1189-YMF-01; Cambio) was diluted in the supplier's hybridization mix. The probe mixture was added to the sections, sealed under glass with rubber cement, heated to 80°C for 10 min, and then incubated overnight at 37°C. The next day, all slides were washed in 50% formamide (284226P; BDH Laboratory Supplies)/2× saline sodium citrate (SSC) at 37°C, then washed with 2× SSC, and then incubated with 4× SSC/0.05% Tween-20 for 10 min at 37°C. Slides were washed and then mounted with glass coverslips using Vectashield hard set mounting medium with 4′,6-diamidine-2′-phenylindole dihydrochloride (DAPI) (H-1500; Vector Laboratories) for subsequent observation under the fluorescent microscope. Slides were analyzed using a Zeiss Axioplan 2 fluorescence microscope (Carl Zeiss UK Ltd., Hertfordshire, UK) equipped with a triple bandpass filter. Images were collected with a cooled charge-coupled device camera (Quantix Corp., Cambridge, MA) and analyzed using Smartcapture 2 software (DigitalScientific Ltd., Cambridge, UK).

### Inflammatory cytokine arrays

To examine if the injection of hUC-MSCs would interfere with the modulation of inflammation after AKI, human and mouse cytokine levels were measured in digested NOD-SCID mouse kidneys which were obtained from the pool of kidneys from six mice per group at day 3. For this purpose, RayBio™ Mouse Inflammatory Antibody Array 1 (RayBiotech, Inc., Atlanta, GA) and Human Inflammatory Antibody Array 3 (RayBiotech, Inc., Atlanta, GA) were used for the protein cytokine assays. Each array was incubated with 250 µg protein extracted from kidney tissues at 4°C overnight, and bound inflammatory cytokines were detected according to the manufacturer's instructions and quantified by densitometry (GS-800 Calibrated Densitometer, Bio-RAD, CA).

### Apoptosis and proliferation measurements

Apoptosis was quantified by a TUNEL assay using an *in situ* cell detection kit (TdT-FragEL™ DNA fragmentation detection kit, Calbiochem, Darmstadt, Germany). TUNEL assays were performed as in our previous studies [44], [45] to detect renal tubular cell death according to the manufacturers' protocol. Color was developed with 3,3′ diaminobenzidine (DAKO, Santa Barbara, CA, USA) and counterstained with methyl green (Vector Laboratories, CA, USA). To compare the TUNEL-positive renal tubular cells in each group, the number of TUNEL-positive cells was counted in 1000 consecutively observed renal tubular epithelial cells per mouse, and the results were expressed as TUNEL positive cells per 10^3^ renal tubular cells.

To determine the number of proliferating renal tubular cells, a PCNA staining kit (No. 93-1143, Invitrogen, San Diego, USA) was used to stain formalin-fixed paraffin renal sections. Briefly, after deparaffination, rehydration, and blocking of slides, the slides were incubated with a biotinylated mouse anti-PCNA primary antibody for 1 h at room temperature, followed by incubation with streptavidin-horseradish peroxidase and treatment with 3,3′ diaminobenzidine substrate (DAKO, Santa Barbara, CA, USA). Nuclei were observed via counterstaining with Harris hematoxylin. In all the experimental groups, the number of PCNA-positive tubular cells was counted in 1000 consecutively observed renal tubular epithelial cells per mouse, and the results were expressed as PCNA-positive cells per 10^3^ renal tubular cells.

### Western blotting analysis

Cells from sonicated renal tissue and hUC-MSCs were lysed on ice with 200 µl of lysis buffer (50 mM Tris-HCl, pH 7.5, 0.5 M NaCl, 5 mM MgCl_2_, 0.5% Nonidet P-40, 1 mM phenylmethylsulfonyl fluoride, 1 µg/ml pepstatin, and 50 µg/ml leupeptin) and centrifuged at 13,000× *g* at 4°C for 10 min. The protein concentrations in the supernatants were quantified using a BSA protein assay kit. Electrophoresis was performed on a NuPAGE Bis-Tris electrophoresis system using 30 µg of reduced protein extract per lane. Resolved proteins were then transferred to PVDF membranes. Membranes were blocked with 5% non-fat milk for 1 h at room temperature and probed with appropriate dilutions of primary antibodies at 4°C overnight: cleaved caspase-3 (Asp175) (1∶1000), cleaved caspase-8 (1∶1000), cleaved caspase-9 (Asp330) (1∶1000), phospho-Akt (Ser473, 1∶1000), Akt (1∶1000), and β-actin (1∶5000), which were purchased from Cell Signaling Technology, Inc. (Danvers, MA), insulin-like growth factor 1 (IGF1)(1∶1000) and IGF1 receptor (IGFR)(1∶1000) (GeneTex, Inc., San Antonio, TX). After the PVDF membrane was washed 3 times with TBS/0.2% Tween 20 at room temperature, it was incubated with an appropriate secondary antibody (goat anti-mouse or anti-rabbit, 1∶10000, Sigma-Aldrich) labeled with horseradish peroxidase for 1 h at room temperature. All proteins were detected using Western Lightning™ Chemiluminescence Reagent Plus (Amersham Biosciences, Arlington Heights, IL) and quantified by densitometry (GS-800 Calibrated Densitometer, Bio-RAD, CA).

### Fluorescent immunohistochemistry for caspase-3, -8, and -9

Renal sections were treated with 3% hydrogen peroxide in 1× PBS for 10 min to block endogenous peroxidase activity after being dewaxed and rehydrated. Next, the sections were washed 3 times with PBS-T (1× PBS containing 0.05% Tween 20) for 10 min each time and non-specific reactions were blocked by 2% BSA in PBS for 1 h at room temperature. Sections were incubated with the first antibodies at 4°C overnight. Sections were washed 3 times with PBS-T (1× PBS containing 0.05% Tween 20) for 10 min each time and then incubated with FITC-conjugated secondary antibody 1 h at room temperature. Sections were then washed with PBS-T 3 times and stained with 300 nM DAPI for 10 min. Images were obtained using a fluorescence microscope (Carl Zeiss, Oberkochen, Germany).

### Statistical analyses

SUN, SCr, TIS, PCNA-positive cells, and TUNEL-positive cell were analyzed by two-way ANOVA for repeated measures (the first factor being treatment group and the second factor being time period) for comparison between groups. When a significant effect was detected by ANOVA, the Newman-Keuls test was used to establish which difference between means reached statistical significance (P<0.05). All data are presented as mean ± SEM.
